# Nanospectroscopy Captures Nanoscale Compositional Zonation in Barite Solid Solutions

**DOI:** 10.1038/s41598-018-31335-3

**Published:** 2018-08-29

**Authors:** Florence T. Ling, Heather A. Hunter, Jeffrey P. Fitts, Catherine A. Peters, Alvin S. Acerbo, Xiaojing Huang, Hanfei Yan, Evgeny Nazaretski, Yong S. Chu

**Affiliations:** 1 Department of Civil and Environmental Engineering, Princeton University, Princeton, NJ 08544 USA; 2 Center for Advanced Radiation Sources (CARS), University of Chicago, Chicago, IL 60637 USA; 3 National Synchrotron Light Source II, Brookhaven National Laboratory, Upton, NY 11973 USA

## Abstract

Scientists have long suspected that compositionally zoned particles can form under far-from equilibrium precipitation conditions, but their inferences have been based on bulk solid and solution measurements. We are the first to directly observe nanoscale trace element compositional zonation in <10 µm-sized particles using X-ray fluorescence nanospectroscopy at the Hard X-ray Nanoprobe (HXN) Beamline at National Synchrotron Light Source II (NSLS-II). Through high-resolution images, compositional zonation was observed in barite (BaSO_4_) particles precipitated from aqueous solution, in which Sr^2+^ cations as well as HAsO_4_^2−^ anions were co-precipitated into (Ba,Sr)SO_4_ or Ba(SO_4_,HAsO_4_) solid solutions. Under high salinity conditions (NaCl ≥ 1.0 M), bands contained ~3.5 to ~5 times more trace element compared to the center of the particle formed in early stages of particle growth. Quantitative analysis of Sr and As fractional substitution allowed us to determine that different crystallographic growth directions incorporated trace elements to different extents. These findings provide supporting evidence that barite solid solutions have great potential for trace element incorporation; this has significant implications for environmental and engineered systems that remove hazardous substances from water.

## Introduction

The precipitation of solid solutions from aqueous solutions has been studied to a great extent due to the prevalence of solid solutions in nature and their importance in engineered systems^1–9^. For example, the plagioclase class of minerals is one of the most abundant solid solutions in terrestrial igneous rocks^10^. Taking inspiration from mineral solid solutions, engineers have applied co-precipitation principles in treating industrial wastewaters produced from oil and gas operations^11–13^, removing contaminants in mine waste^14–16^, and in the storage of radioactive waste^6^. In analytical chemistry, the need to synthesize pure chemicals has led to efforts to limit trace element incorporation during precipitation^17,18^.

Despite the widespread occurrence of solid solutions, their formation is poorly understood. Thermodynamic models have been extensively developed^9,19,20^, but kinetically controlled processes during precipitation limit the accuracy of thermodynamic predictions^21–23^. When kinetic processes are limiting and the reaction has not achieved equilibrium, heterogeneity in the surrounding fluid can occur, which can then lead to spatial heterogeneities within a solid solution^2,24^. This is referred to as compositional zonation. In analytical chemistry, chemists predict the existence of compositionally zoned particles as they precipitate from poorly-mixed, heterogeneous solutions. In geologic systems, compositional and sometimes oscillatory zonation occurs, for instance, in the plagioclase solid solution series, which includes NaAlSi_3_O_8_ and CaAl_2_Si_2_O_8_, and is interpreted as the outcome of out-of-equilibrium conditions that preferentially partitions certain elements into the crystal from the fluid phase^25^. Compositional zonation can also act as a record for the growth history of a crystal, documenting changes in the fluid chemistry, pressure, or temperature conditions in the surrounding environment. In engineered systems, compositional zonation serves as an indicator of the potentially large amounts of trace elements that can be taken out of solution, if the conditions are favorable.

The relationship between trace element incorporation into solid solution and whether it manifests as compositional variation is complex, with several models developed to predict the possible outcomes. For solution conditions that promote rapid precipitation and compositionally zoned particles, the nonequilibrium partitioning law can be used to predict the extent of trace element incorporation into the solid phase^1,24^. In contrast, the homogeneous partition law is used for near-equilibrium conditions in which homogeneous particles are expected to precipitate^6,24,26,27^.

Pina and Putnis^28^ have also developed a model to predict trace element incorporation and compositional zonation, incorporating theory for the nucleation and growth of solid solutions. Their modeling results have been compared to counterdiffusion experiments, in which solutes in two aqueous solutions diffuse through a porous silica hydrogel before reacting to form zoned particles. In these experiments, Putnis *et al*.^29^ and Prieto *et al*.^30^ successfully produced patterns of compositional and/or oscillatory zonation for (Ba, Sr)SO_4_ and (Cd,Ca)CO_3_ solid solutions. According to Prieto *et al*.^9,30^, the solubility constants of the solid solution endmembers must differ by several orders of magnitude. In other work^31–33^, modeling of crystal growth at the mineral-fluid interface supports the hypothesis that compositional zonation is promoted by a difference in the endmember solubility constants of several orders of magnitude, combined with diffusion-limited solute transport at the mineral-fluid interface. In the example of (Ba, Sr)SO_4_, the solubility product constant (K_sp_) values of endmembers BaSO_4_ (pK_sp_ = 9.98)^34^ and SrSO_4_ (pK_sp_ = 6.63)^35^ differ by three orders of magnitude. Because BaSO_4_ is less soluble than SrSO_4_, it is expected for BaSO_4_ to precipitate out of solution first. When Ba^2+^ is sufficiently depleted at the mineral-fluid interface due to incorporation into the solid phase as BaSO_4_, Sr^2+^ can then be preferentially incorporated until an influx of diffusing Ba^2+^ ions can replenish the supply for further BaSO_4_ precipitation. In counterdiffusion experiments, these effects produce >40 µm sized particles with ~5 to ~10 µm wide bands of high Sr^2+^ content after reacting for ~1 month^29,30,36^.

While counterdiffusion experiments enable control of solute mass transfer and unique inferences about controls on zonation, precipitation of particles directly from an aqueous solution represents reality for most natural and engineered systems. Among these systems, solution conditions can vary widely from freshwater to saline environments. Consequently, one may need to account for deviations from ideal solution conditions to accurately predict trace contaminant incorporation into solids, a process that is often hindered by the need for accurate solution and solid-phase activity coefficients which often require fitting of additional experimental data^24,37^. For solid-phase activity coefficients, methods based on estimation of enthalpic and entropic contributions to excess free energy^38,39^ have also been used to derive the necessary parameters to calculate solid-phase activity coefficients.

Studying compositional zonation and variation in particles precipitated from aqueous solutions is also challenging because they can produce micron to nanosized particles. Due to the small size of the particles, experimental methods have been limited to bulk solid and solution analyses. Until now, solid phase X-ray fluorescence (XRF) imaging has not been applied to study any nanoscale chemical features that may be present in small particles or the colloidal fraction of solids precipitated from aqueous environments.

For the first time, we have observed nanoscale compositional zonation in <10 µm sized barite particles precipitated from an aqueous solution within minutes at ambient temperature and pressure. In two sets of experiments, we examined incorporation of Sr^2+^ (substituting for Ba^2+^) in BaSO_4_ and incorporation of HAsO_4_^2−^ (substituting for SO_4_^2−^) in BaSO_4_, each at a high and a low salinity condition. Barite precipitation from aqueous solution has broad relevance, including use of Na_2_SO_4_ to induce barite precipitation for removal of metals from industrial wastewaters^11,12,40,41^. We found that compositional zonation occurred even when one of the solid solution endmembers remained below saturation throughout the course of the experiment. This contrasts with experiments by Prieto *et al*.^30^ in which both endmembers were supersaturated when precipitation occurred, although theory^9,28^ does not require that both end-members need to be supersaturated in order to produce compositional zonation. Furthermore, we found that zonation was promoted by high salinity conditions.

The discovery of nanoscale zonation in precipitated particles was made possible with the advent of multimodal capabilities at the Hard X-ray Nanoprobe (HXN) beamline 3-ID at the National Synchrotron Light Source-II (NSLS-II) of Brookhaven National Laboratory (Upton, NY). Recent advancements have improved the spatial resolution and sensitivity of X-ray spectroscopic methods to enable imaging within structures, ranging from metal nanoparticles within bacterial cells^42^ to contaminants within complex sediments^43^. High resolution XRF imaging is particularly challenged by the need to focus X-ray beams at sufficiently high energies to apply nanospectroscopy^43^. The properties of the X-ray source at NSLS-II and the use of Multilayer Laue Lenses (MLL) at the HXN beamline^44,45^ provide the necessary specificity, spatial resolution, and sensitivity to make detailed mapping of single particles possible. Owing to the high-precision of the HXN X-ray microscope, we were able to overcome considerable complexities of adjusting the focal length of a set of MLLs required for a nanospectroscopy experiment^46^. The HXN can produce nano-XRF maps with a spatial resolution down to 15 nm^45,47^. Differential phase-contrast (DPC) imaging, performed in parallel with XRF imaging, provides morphology mapping that is sensitive to the electronic density of the sample^46,48^. For this study, individual crystals, many of which were <10 µm in size, were mapped to examine the spatial distribution of elements within the particles. The ability to conduct high-resolution imaging and elemental mapping of precipitated particles from aqueous solutions will extend the existing theories of solid solutions and trace element co-precipitation by enabling direct analysis of solid-phase compositional variation.

## Results

### Nanoscale compositional zonation in particles precipitated from aqueous solutions at high salinity

In this study, incorporation of trace elements into barite was quantified using fractional substitution of the trace ions. The fractional substitution of the cation Sr^2+^ for Ba^2+^ is quantified as:

1 $${f}_{S{r}^{2+}}=\frac{{n}_{Sr}}{{n}_{Sr}+{n}_{Ba}}$$

where *n*_*i*_ is the moles of element *i*. As described in the Methods section, it was not possible to quantify in absolute terms of the moles of each element, but it is possible to quantify the relevant ratios, and because both Sr and Ba are strongly detected at the HXN beamline, the ratio in equation 1 is directly quantified from the relevant X-ray fluorescence intensities. For Arsenic, HAsO_4_^2−^ ions substitute for SO_4_^2−^ in barite^49,50^. Therefore, the fractional substitution of the anion HAsO_4_^2−^ for SO_4_^2−^ is represented as:

2 $${f}_{HAs{O}_{4}^{2-}}=\frac{{n}_{As}}{{n}_{As}+\,{n}_{S}}$$

Because it was not possible to reliably detect Sulfur via XRF at this beamline, we quantified this fractional substitution as the ratio of moles of As to Ba. This ratio is the same as the fractional substitution in equation 2, by the stoichiometry of the solid solution Ba(SO_4_)_x_(HAsO_4_)_1−x_.

**Figure 1:**
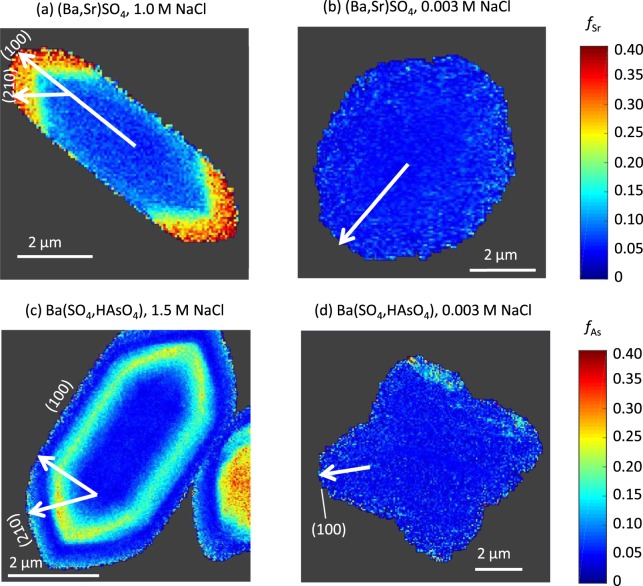
The fractional substitution maps for selected particles from the (Ba,Sr)SO_4_ experiments, at (**a**) 1.0 M NaCl, or (**b**) no additional NaCl. The fractional substitutional maps for the Ba(SO_4_,HAsO_4_) experiments, at (**c**) 1.5 M NaCl, or (**d**) no additional NaCl. Vectors are drawn for crystal growth directions towards the (100) or (210) surfaces, although morphology and crystal planes could not be determined for the particle displayed in (**b**).

**Figure 2:**
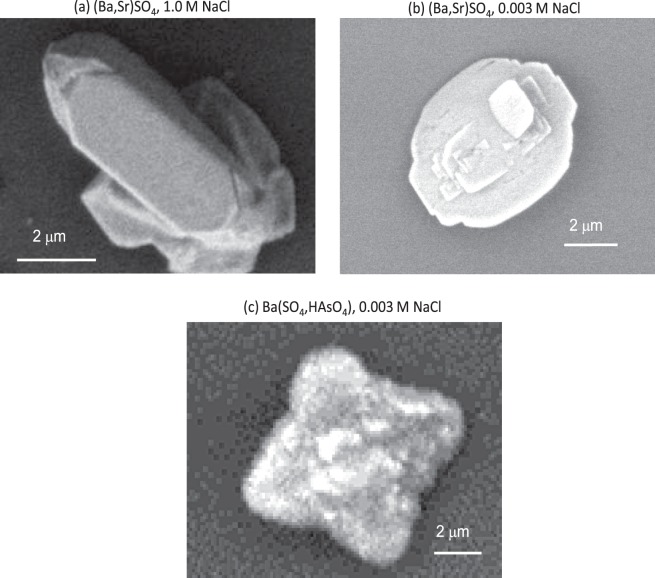
SEM images of particles displayed in Fig. (**a**) 1a and (**b**) 1b taken on a Quanta 200 FE-ESEM, and of (**c**) 1d, taken on a JEOL JCM-6000 NeoScope.

**Figure 3:**
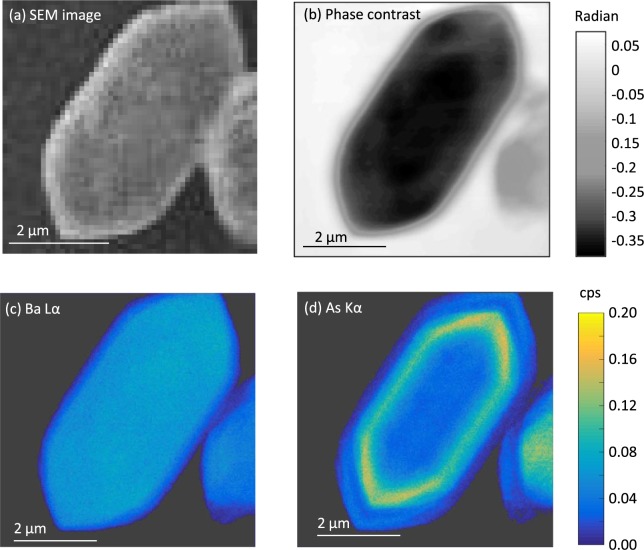
For a selected Ba(SO_4_,HAsO_4_) particle, (**a**) an SEM image, (**b**) the phase contrast image, (**c**) the Ba Lα and (**d**) the As Kα maps in counts per second (cps) normalized to the ion chamber (I_0_) reading.

The fractional substitution values were used to generate image maps for several particles from each of the four experimental conditions, and representative examples have been selected for Fig. 1. Associated SEM images are shown in Figs 2 and 3a. Due to the small thicknesses of the particles in the *z* direction (~0.1 μm), the synchrotron X-rays fluoresce the entire volume of the sample at a single point, leading to observation of compositional zonation only in the *x-y* plane. In the experiments with no added NaCl, trace element incorporation was consistently small, with fractional substitutions around 0.05 for both Sr and As. Furthermore, no compositional zonation was observed at low salinity (Fig. 1b,d).

At high salinity, compositional zonation was consistently observed. In the 1.0 M NaCl condition for (Ba,Sr)SO_4_ (Fig. 1a), particles containing a Sr-rich edge were consistently found. The *f*_*Sr*_ was ~0.06 in the center of the particle, when the particle was first forming, increasing to ~0.34 as the particle grew. Similarly in the 1.5 M NaCl condition for Ba(SO_4_,HAsO_4_), As-rich zones were frequently found. In the selected particle in Fig. 1c, the As-rich zone is ~0.2 to 0.75 µm wide. The *f*_*As*_ was ~0.06 in the center of the particle, during initial particle formation, and increased to ~0.33 as the particle grew. Interestingly, for the particle shown in Fig. 1c the amount of As incorporation decreased back to ~0.06 on the particle edge near the end of the particle’s growth period, displaying oscillatory zonation.

### Novel multimodal capabilities for nanospectroscopy

The multimodal capabilities to combine quantitative high resolution elemental mapping, phase contrast imaging, and scanning electron microscopy were instrumental in this study. Figure 3 showcases the power of combined imaging modalities. For the same selected particle from the Ba(SO_4_,HAsO_4_) experiment that was depicted in Figs 1c and 3a displays a SEM image. SEM was used to identify particles that were isolated and lying flat. SEM was also essential for locating particles relative to the navigational Pt grid of the sample board.

Figure 3c shows a map of the emission intensity of Ba Lα, and it illustrates the uniform presence of Ba throughout the particle. Figure 3d shows a map of the emission intensity of the As Kα, and it displays a pattern consistent with the compositional zonation observed in the fractional substitution map in Fig. 1c. In each of these maps, of note is the lower emission intensity within ~0.2 µm of the perimeter of the particle. This is also observed in the As Kα intensity map (Fig. 3d). Examination of the phase contrast image of the same particle (Fig. 3b) guards against misinterpretation, revealing that the reduced emission intensities are due to thinning of the particle at the edges. The phase contrast image also provides evidence supporting the observation of compositional zonation of HAsO_4_^2−^ incorporated into barite. Where the banded As-rich region is evident in Fig. 1c, the phase contrast map exhibits a medium gray shade, contrasting with the dark region in the particle center where SO_4_^2−^ dominates. In a phase contrast image, a darker region is caused by the presence of heavier elements, a thicker area, or a higher density^51^. The SEM image does not show significant variation in surface morphology (i.e. height variation) between the two regions (Fig. 3a), suggesting that the differences in grayscale in the phase contrast region are not caused by differences in thickness in the particle. This is also supported by the uniformity of the particle in the Ba Lα XRF image (Fig. 3c). Additionally, HAsO_4_^2−^ has a higher molar mass than SO_4_^2−^, which should result in the As-rich region being darker than the As-poor center. However, Fig. 3b indicates that the As-rich region is lighter than the SO_4_^2−^ rich particle center. Consequently, we conclude that the As-rich region has a lower density than the center of the particle, overriding the effects of differences in molar mass. Supporting our interpretation, Ma *et al*.^50^ show through analysis of X-ray diffraction data that the incorporation of As(V) into the barite structure during co-precipitation expands the unit cell volume, which can subsequently decrease the density.

**Figure 4:**
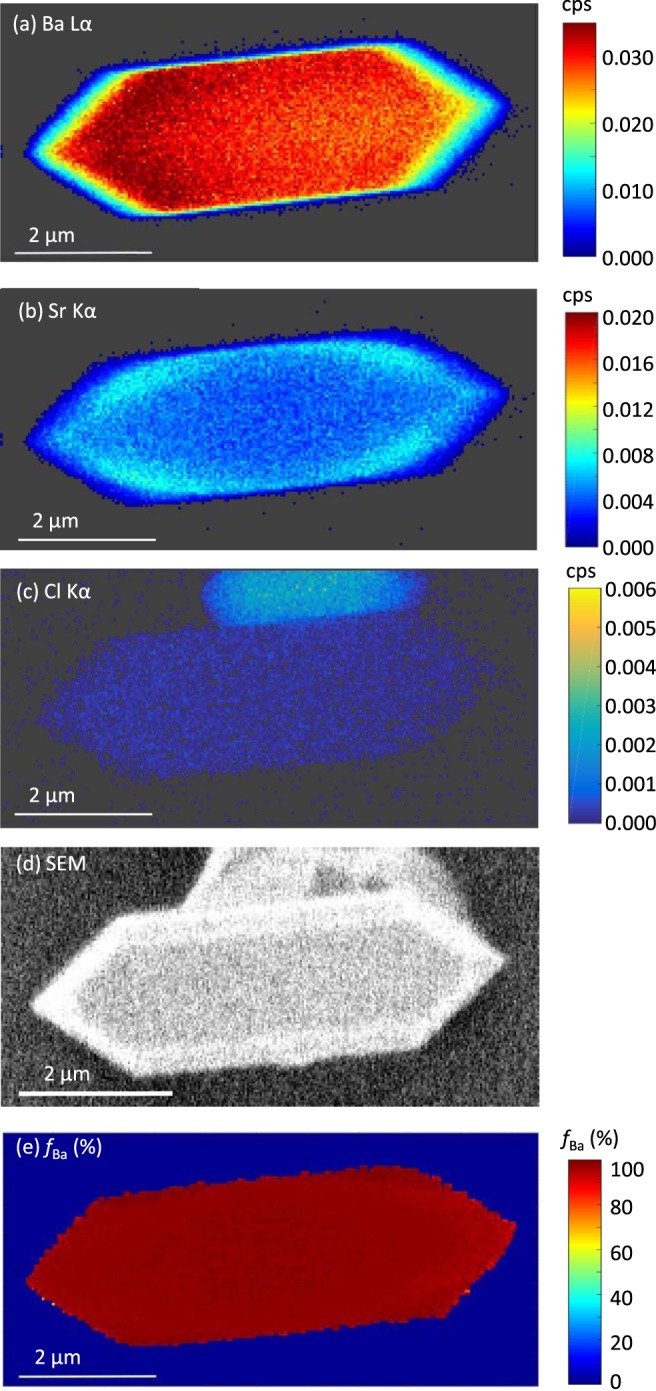
For a selected (Ba,Sr)SO_4_ particle, maps of emission intensities for (**a**) Ba Lα, (**b**) Sr Kα, (**c**) Cl Kα in counts per second (cps) normalized to ion chamber (I_0_) reading. Also shown are (**d**) the SEM image, and (**e**) the calculated Ba fractional substitution.

The importance of quantitatively interpreting the XRF data is demonstrated in multimodal images for the particle from the (Ba, Sr)SO_4_ experiment (Fig. 4). The XRF intensities for Ba Lα (Fig. 4a) and Sr Kα (Fig. 4b) might suggest a zone with lesser Ba around the edge. However, the SEM image (Fig. 4d) shows particle faceting at the edge. This would reduce the Ba Lα and Sr Kα signals. When the raw data are processed to calculate the *f*_*Ba*_, the apparent compositional zonation disappears, revealing a uniform Ba-rich particle (Fig. 4e). Quantification of the extent of trace element substitution can eliminate misinterpretation of the edge effects observed in the raw XRF data when the crystals exhibit facets near the edges.

Another interesting example of the power of multimodal imaging is the ability to distinguish salt particles. As shown in the SEM image in Fig. 4d, two crystals are together, one atop the other. From the map of emission intensities for Cl Kα (Fig. 4c), it is evident that the one underneath is salt rather than barite. There is a weak Cl Kα signal within the (Ba, Sr)SO_4_ particle, likely due to interference from other peaks during fitting of the XRF spectrum.

The multimodal capabilities at the HXN allow us to reliably identify chemical compositional zonation and differentiate it from observations caused by physical characteristics of the particles. Quantitative analyses of the raw XRF maps confirm that the observation of nanoscale compositional zonation stems from different amounts of trace element incorporation during co-precipitation, as opposed to changes in crystal faceting or texture. SEM images and phase contrast imaging provided additional support for such a conclusion.

### Barite growth history recorded in a single particle

**Figure 5:**
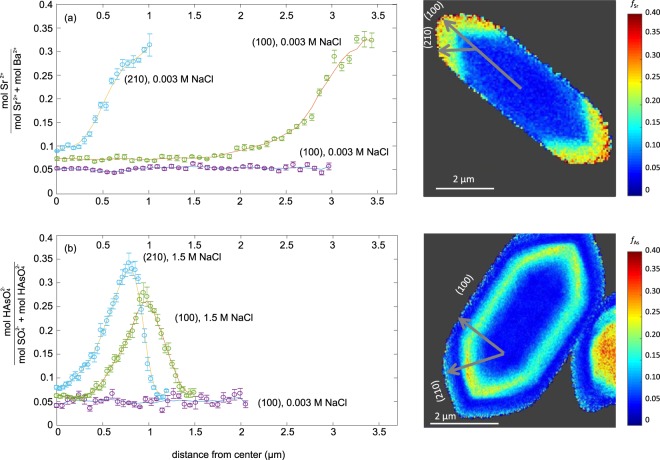
Profile plots of the fractional substitution of (**a**) Sr^2+^ in a selected particle from the (Ba,Sr)SO_4_ experiments, and (**b**) HAsO_4_^2−^ in a selected particle from the Ba(SO_4_,HAsO_4_) experiments along the transects corresponding to crystal growth directions indicated in the fractional substitution maps on the right for particles from experiments at high salinities. The growth directions for experiments at low salinity are indicated in Fig. 1b,d.

The illuminating power of nanoscale spatially resolved element quantification of individual particles is demonstrated by examining fractional substitution profiles along transects aligned with the different crystal growth directions. Figure 5 presents Sr and As fractional substitution profiles along growth directions depicted in Fig. 1 towards the (100) and (210) crystal planes, respectively. The degree of saturation in solution, the cation and anion ratios, the presence of NaCl, and the presence of various organics can all influence the growth rates of different crystal faces and therefore the resulting particle morphology^13,52–55^.

For the high salinity experiments, in which little trace element incorporation was observed at the start of crystal growth, the relatively homogeneous centers varied by <1% Sr or As (Fig. 5). The fractional substitution values for experiments with 0.003 M NaCl remain close to the particle centers for experiments with high NaCl. When trace element incorporation began to increase from the baseline for experiments at high NaCl, for the Sr particle, similar amounts of trace element incorporation were achieved towards both the (210) and (100) planes (Fig. 5a). In contrast, for the As particle, the fractional substitution of As peaked at 27% in the direction towards the (100) plane, and at 34% As in the direction towards the (210) plane (Fig. 5b). Differences in trace element incorporation between two growth directions may be caused by the differences in bonding environment for each respective crystal surface. The density of Ba^2+^ and SO_4_^2−^ bonding differ for these two faces, with the (210) surface having 8 Ba-O broken bonds per unit cell, while the (001) surface has 4 broken Ba-O bonds per unit cell^56^. This results in different terminations of water molecules on each surface, and likely explains the differing growth rates between the two crystal faces.

## Discussion

This study showed that Sr and As co-precipitation in (Ba,Sr)SO_4_ and Ba(SO_4_,HAsO_4_) solid solutions (respectively) is favorable under high salinity conditions and that it produces nanoscale compositional zonation in particles as small as 10 μm. This is the first nanospectrocopy investigation using MLLs. These first-time observations were also made possible because of the multimodal capabilities of the HXN X-ray microscope which enabled parallel imaging using SEM and phase contrast. The ability to interpret the XRF maps in terms of elemental ratios further enabled quantitative inferences about trace element incorporation in solid solution.

We attribute the observation of compositional zonation in barite to the effects of salinity on the thermodynamic driving force for precipitation and the rates of nucleation and growth. NaCl has a strong influence on species activity and therefore on the thermodynamic driving force. The saturation index (SI) of an aqueous solution is a measure of the extent to which the solution deviates from the equilibrium with the mineral:

3 $$SI=log\frac{\{A\}\{B\}}{{K}_{sp}}$$

where {*A*} and {*B*} are the activities of the cation and anion respectively for the mineral of interest, and *K*_*sp*_ is the solubility constant. In the Sr experiments in which concentrations of Ba^2+^, SO_4_^2−^, and Sr^2+^ were the same but salinity was different, the BaSO_4_ SI was 3.9 in the (Ba,Sr)SO_4_ experiments at low salinity, and 2.5 at 1.0 M NaCl. Similarly, in the As experiments, the BaSO_4_ SI was 3.7 in the Ba(SO_4_,HAsO_4_) experiments at low salinity, and 0.1 at 1.5 M NaCl. The SI values for all solid solution endmembers are listed in Table 1. The lower SI values result from reduced ion activities from increased ionic strengths. (Ion activities were calculated from PHREEQC^57^ using the Pitzer database, with supplementary data from Millero and Pierrot^58^).

**Table 1:** Saturation indices for solid solution endmembers.

(Ba,Sr)SO_4_		
NaCl (M)	${{\bf{S}}{\bf{I}}}_{{{\bf{BaSO}}}_{{\bf{4}}}}$	${{\bf{S}}{\bf{I}}}_{{{\bf{SrSO}}}_{{\bf{4}}}}$
0.003	3.9	0.1
1.0	2.5	−1.2
**Ba(SO**_**4**_ **,HAsO**_**4**_ **)**		
**NaCl (M)**	${{\bf{S}}{\bf{I}}}_{{{\bf{BaSO}}}_{{\bf{4}}}}$	${{\bf{S}}{\bf{I}}}_{{{\bf{BaHAsO}}}_{{\bf{4}}}}$
0.003	3.7	−1.3
1.5	0.1	−4.2

The pK_sp_ values of BaSO_4_^34^, SrSO_4_^35^, and BaHAsO_4_^65^ are 9.98, 6.63, and 5.60, respectively.

Negative values of SI represent thermodynamic conditions that would typically favor dissolution rather than precipitation. In our high salinity experiments, both SrSO_4_ and BaHAsO_4_ are undersaturated and would be expected to dissolve (Table 1), but as a solid solution, it is possible to precipitate components of the undersaturated endmembers (Fig. 1). This behavior likely stems from differences in the SI values of the solid solution endmembers in comparison to measures of supersaturations for intermediate solid solutions, such as total solubility product^37^ or supersaturation functions^20,28^.

Because NaCl influences the thermodynamic driving force behind precipitation, it subsequently controls the kinetics of solid nucleation and growth that will determine the extent to which concentration gradients within the fluid phase occurs. For instance, it is well established that a decrease in the SI at constant concentration can slow nucleation and growth^18^, which would tend to minimize the establishment of chemical gradients between the solid surface and the bulk solution and would consequently produce a homogeneous particle. However, excessively fast nucleation and growth can also produce uniform particles if the most recent layer of precipitation deposited on the crystal surface does not have sufficient time to equilibrate with localized solution conditions from the chemical gradients formed during rapid precipitation. Compositional zonation, according to the nonequilibrium partition model, is expected only if precipitation rates are *high* enough to prevent recrystallization during crystal growth, but *low* enough to allow incremental solid-phase layers to equilibrate with the solution during the time of their formation^24^. Therefore, to produce compositional zonation in our experiments, the NaCl must decrease the nucleation and growth rates just enough such that the incremental solid-phase layers have time to equilibrate with the solution, producing nanoscale compositional zonation of Sr or As, but not so much that it allows for true equilibration of the entire particle through recrystallization. In contrast, we expect that the experiments with no additional NaCl had nucleation and growth rates that were too rapid to allow for equilibration of the precipitating incremental surface.

In addition to its effect on the thermodynamic driving force in slowing nucleation and growth, NaCl may also increase nucleation and growth kinetics by modifying the hydration of ions in solutions. Background electrolytes can diminish ion hydration, removing water molecules that shield the nucleation and growth of barite. Consequently, at a constant saturation, increasing ionic strength has been shown to increase growth rates^59,60^. Risthaus *et al*.^59^ suggest that Na^+^ may even attach to growth surfaces on barite, starting a new row of growth. Because our experiments vary both saturation and salinity, NaCl may play multiple roles in altering nucleation and growth rates to produce conditions that allow for compositional zonation.

A third possibility of the effect of NaCl involves the role in forming nanoscale fluid inclusions in the barite particles. Weber *et al*.^61^ found that barite particles equilibrated in a NaCl solution developed regions with small and large nanopores alternating in a laminar pattern. When these particles were immersed in a Ra-containing solution, the nanopores acted as pathways for increased Ra uptake^62^. A similar phenomenon involving the development of nanoporosity and increased trace element uptake may be occurring in our high salinity experiments.

The presence of a Sr-rich or As-rich zone that is ~3.5 to ~5 times more highly concentrated in the trace element than the particle center suggests strategies for engineered systems of co-precipitation for trace contaminant removal. These results suggest that removal of As and Sr, and by extension other trace elements, can be done efficiently at high salinities, even if the solution is undersaturated with respect to the substitutional end-member. The remaining challenge is to identify the surface chemistry conditions that fostered high trace element incorporation, and to use this knowledge to control the rates of precipitation in an engineered system to maximize contaminant removal.

With the ability to observe and quantify the amount of trace elements incorporated into small particles directly precipitated from aqueous solutions, this study provides supporting evidence for current theory previously supported only by bulk solid and solution measurements, and hydrogel diffusion experiments. Developing a well-established theory that can predict kinetically-controlled co-precipitation can reduce the need for experimentally derived partition coefficients, and enable improved modeling for solid solution formation in a wide range of systems.

## Methods

### Precipitation experiments

Precipitation experiments were conducted by starting with BaCl_2_ solution in standard disposable cuvettes for ultraviolet spectroscopy. All at once, reactant solutions containing Na_2_SO_4_ and either SrCl_2_ or Na_2_HAsO_4_ were added. NaCl was added as needed to produce the desired mixture concentration. All stock chemicals were purchased from Fisher Scientific except for 99% SrCl_2_ • 6H_2_O, which came from Arcos. For Sr co-precipitation experiments, the resulting concentrations were 1.5 mM BaCl_2_, 1.5 mM Na_2_SO_4_, and 0.5 mM SrCl_2_, and the final NaCl was set at ~0.003 or 1.0 M. For As co-precipitation experiments, the resulting concentrations were 1.4 mM BaCl_2_, 1.4 mM Na_2_SO_4_, and 0.45 mM Na_2_HAsO_4_, and the final concentration of NaCl was set at ~0.003 or 1.5 M. Experiments were sampled 0.5 to 2 hrs after mixing, guided by ultraviolet absorption as an indication of particles forming and settling.

### Sample mounting and characterization

Drops of ~1 μl sample were placed on Norcada mounting chips, which are 3.5 mm × 1.5 mm × 300 μm cantilevered silicon wafers with an imprinted Pt grid to enable navigation. The drops were allowed to dry to deposit barite particles. Samples from experiments at high salinity were gently rinsed in Milli-Q water to dissolve salt crystals. At the beamline, mosaics of SEM images of the mounted samples were then collected (JEOL JCM-6000 NeoScope) to map particles of interest for XRF imaging. For select samples, additional high resolution SEM images were collected on a Quanta 200 FE-ESEM.

### X-ray nanospectroscopy imaging

The MLL with nanofocusing optics resulted in a 12 × 13 nm focused beam. A three-element silicon drift detector (SDD, Vortex), positioned perpendicular to the X-ray beam, was used for collecting XRF spectra. A pixel array detector (512 × 512 pixels and 55 um/pixel, Merlin), positioned at 0.5 m downstream of the sample, was used for DPC imaging, which is based on the transmitted intensity of the X-ray beam, and was used to map phase density and constrain particle thickness. For XRF imaging, raster scanning was used by continuous fly-scanning across individual barite particles at 30 to 60 nm steps with dwell times from 100 to 250 ms. For Sr, XRF spectra were collected at 16.118 keV. For As, XRF spectra was collected at 11.874 keV. These energies were selected in order to increase sensitivity for detecting the elements of interest.

Spectral fitting of XRF data was performed using PyXRF^63^ in order to quantify emission intensities and create maps of S Kα, Ba Lα, Sr Kα and As Kα. Custom Matlab (Mathworks) code in conjunction with NIST NRLXRF software (Birks *et al*. 1977) was used to interpret emission intensities (cps), and ultimately compute the fractional substitutions for Sr and As. For this, particle thickness was estimated based on particle aspect ratios in SEM images. NRLXRF, which accounts for X-ray fluorescence escape depth, demonstrated the relative insensitivity of the quantitative XRF results to sample thicknesses ranging from 0.05 to 5 μm. Two calibration curves were generated assuming (Ba, Sr)SO_4_ and Ba(SO_4_, HAsO_4_) solid solution stoichiometry. The theoretical fluorescence intensities of the Ba Lα, Sr Kα and As Kα emission lines were computed using NRLXRF for different amounts of Sr or As ranging from no substitution to complete substitution. Uncertainties would make it difficult to accurately quantify the absolute amounts of each element. However, the ratio of two emission intensities, such as Ba Lα to As Kα, can be computed accurately. This was verified by XRF imaging particles of pure BaSO_4_ and SrSO_4_ standards. Experimental values of the Sr Kα/(Ba Lα + Sr Kα) ratios were found close to 0 and 1, respectively. For the Sr experiments, strong XRF signals of both elements (Ba and Sr) were obtained. For the As experiments, the weak signal for S did not allow quantification. To work around this, a calibration curve was constructed using a power law fit to the ratio of theoretical emission intensities for Ba Lα/As Kα for a range of As fractional substitution values. Finally, for the experimental samples, the two calibration curves were used to compute Sr or As fractional substitution values from the raw measured emission intensities using a spline fit to the respective calibration curve.

Particle profiles were created from the fractional substitution maps by drawing neighboring transects from the center to the particle edge in along the crystal growth directions indicated in Fig. 1. The crystal planes and growth directions are labeled depending on the barite morphologies depicted in Goldschmidt^64^. They were identified as barite no. 12, 35, and 62 for the particles shown in Fig. 1a,c,d, respectively. The morphology of the particle shown in Fig. 1b could not be identified. The fractional substitution values from six neighboring transects were averaged, and the values were used to calculate error bars.
